# Supplementary material for: Heparan sulfate attachment receptor is a major selection factor for attenuated enterovirus 71 mutants during cell culture adaptation
Source: PLoS Pathog. 2020 Mar 18;16(3):e1008428. doi: 10.1371/journal.ppat.1008428 (PMC7105141; doi:10.1371/journal.ppat.1008428)
Supplement: Attachment — Submitted filename: Response to revisers PPATHOGENS-D-19-01860 final.docx [file ppat.1008428.s001.docx]

**Part I - Summary**

Reviewer #1: This manuscript by Kobayashi et al details the fitness of the “virulent” EV71 is low in the cell culture environment. Two major adaptive mutants located in VP1-145G or VP1-145Q were produced through the error-prone RNA replication process and can replicate in the cultured cells and eventually become fixed in the population. Interestingly, both of the cell cultured adaptive mutants are attenuated virus when they were tested for virulence using hSCARB2 mice, indicating that avirulent mutants were rapidly selected in the cultured cells, but not survival in the nature host. Although the authors only used the RD cells for the selection of VP1-145 mutants, the author clearly demonstrated the attenuation of EV-A71 during the cell culture adaptation is exclusively associated with VP1-145 mutation. Scientifically speaking, the design of the entire manuscript is clear and the experimental results are correct. However, the phenomenon pointed out in the EV-A71 cell cultured adaptive mutants does not occur in nature host. While these mutants could be applied for vaccine development is still question.
All of the experimental results are convinced and statistical analyses are clearly shown. However, the conclusions of this manuscript may lead to a confused and it will be a fallacy for virologists

We considered why the Reviewer #1 felt our conclusion confusing. We finally found that our description was not kind enough to explain the situation of EV71 propagated in cell culture. When we isolated EV71 from clinical samples of HFMD patients, the CPE does not appear quickly but several blind passages are sometimes required before the CPE appears. This is actually an adaptation process. However, many researchers handle with EV71 that had been already adapted to cultured cells. Therefore, RD cells and Vero cells are commonly used for the propagation of EV71 and this is recognized as a reasonable method. However, in general, a propagation of a virus isolated from animals always has a potential problem that the culture condition may not be the natural condition and it places a stress on the virus. Our result suggests that EV71 is exposed to such severe condition (this was not clearly described in the original version of the manuscript) and that the virus has to overcome this load. Acquiring HS binding capacity and new infection pathway is a viral strategy to survive the severe cell culture condition. We added this explanation in author summary (**lines 46-48**), introduction (**lines 81-82**), results (**lines 141-150, 223-229, 282-287, and 292-294**), and discussion sections (**lines 427-430 and 436-437**). In the revised manuscript, the Reviewer #1 is able to know that this manuscript describes the molecular mechanisms of this adaptions and our conclusion is not a fallacy for virologists.

As written in the response to your specific comment, we confirmed our proposed model using two additional cell lines including Vero and HeLa-S3 cells. The revised manuscript includes the data of VP1-145 mutation in Vero and HeLa-S3 cells in **Figure 1 and 2**. We believe this modification strengthen our conclusion.

Various live attenuated vaccines have been established by cell culture adaptation as described in **lines 497-500**. This work showed an example of attenuation mechanism associated with cell culture adaptation. We are not going to claim this mutation is suitable for live attenuated vaccine of EV71. We did not modify the text regarding with this subject.

Reviewer #2: In this manuscript, Kobayashi et al. elucidated the population dynamic of EV71 during adaptation in cell culture system using NGS analysis. They found a VP1 E145G/Q substitution occurred during virus passaging in RD-A cells. This amino acid substitution is known to enhance HS binding and make virus attenuated. They also found that the population of VP1-145E virus was stable by culturing in HS deficient and hSCARB2 overexpressing (deltaEXT+hSCRB2) RD-A cells; however, VP1-145G and Q viruses gained mutations in both RD-A and deltaEXT+hSCRB2 cells. The VP1-145G/Q mutations further promoted to acquire secondary amino acid substitutions on P1 region, and these mutations are near the 5-fold axis, which is critical for virus binding to cellular receptors. VP1-145G/Q viruses had higher infectious efficiency compared with VP1-145E virus, and the infectious efficiency in deltaEXT+hSCRB2 cells was higher among VP1-145E, G and Q viruses compared to that of RD-A cells. Altogether, the authors explore a possible mechanism of EV71 adaptation and attenuation in cell culture system. The attachment receptor HS, plays an important role for selection VP1-145 mutation and other accessory mutations on EV71 virus which results in avirulent and attenuated viruses.

**Part II – Major Issues: Key Experiments Required for Acceptance**

Reviewer #1: 1. Only one passage: VP1145 E to G in 2716-Ymg-03 strain; while VP1-145 E to Q in Isehara strain, what is the major “pressure” to cause this mutation?

It might be very surprising for the Reviewer #1 to know that virus population almost completely changed in only one passage. This was because infection of VP1-145E virus cannot infect efficiently in cultured cells. This is really a serious bottleneck for the virus. Our data presented two important selection pressures: low surface expression of hSCARB2 and high surface expression of HS. The former pressure makes infection efficiency of EV71 low in cultured cells. The latter pressure selects HS-binding (VP1-145G and Q) mutants. This model was experimentally confirmed because this selection was not observed in RD-∆EXT1+hSCARB2 cells, but in RD-∆EXT1 and RD+hSCARB2 cells (Fig 3-5). To clarify this point, we added explanations in **lines 46-48, 81-82**, **141-150, 223-229, 282-287, 292-294**, **427-430, and 436-437**.

Reviewer #1 may want to know whether the VP1-145G virus and VP1-145Q virus are selected by its specific selection pressure or not. We do not have evidence that there is a specific pressure for each mutation. Both E to G and E to Q mutations occur as a result of single nucleotide mutation of the second and the first letter of glutamic acid (GAG to GGG and GAG to CAG), respectively. We demonstrated the evidence of stochastic selection of VP1-145G and VP1-145Q mutants in RD-A cells. In Figure 5A, Isehara-E virus was passaged in RD-A cells. After three passages of the 5 independent passage experiments, we found two VP1-145G-dominant virus populations and three VP1-145Q-dominant ones (**lines 287-290**). This data indicated that these two mutants were occurred by chance and we speculated that a mutant first emerging in the population should be selected as a dominant mutation. We added the explanation in **lines** **439-441**.

2. What is the main reason that avirulent mutants are rapidly selected in the “RD” cultured cells? How about in the other cell line? Is there any cell tropism issue to cause this phenomena?

HS binding mutants (VP1-145G or Q) are selected in cell culture because it is important for the survival in this condition. This selection was done completely independent of pathogenesis *in vivo*. RD cells does not select viruses that exhibit attenuated phenotype but the virus that can survive in the given condition. The reason for the selection of HS binding mutants are already described.

We have already confirmed the generality of VP1-145 mutation using other cell lines including Vero and HeLa-S3 cells. We added the data to **Figure 1C-D, 1G-H, and 2A** and description of this result in **lines 202-210 and 528-531**.

This selection is associated with cell tropism issue. HS is expressed ubiquitously but the expression level of HS in each cell line is different. Thus, in theory, the susceptibility of cells to the HS binders may depend on the expression levels of HS. However, we have not yet observed such example.

3. VP1-145 is a very important amino acid for virulence in mice. But, what is the role in the cultured cell?

As written in the response to the summary of Reviewer #1, EV71 circulating in human population hardly infect to cultured cells. VP1-145 mutations confer the ability for efficient replication in cultured cells to the virus. This is very important for the survival of the virus.

4. VP1-145G and Q mutations also modulate infection efficiency, do these mean that this amino acid will interact with many host factors?

We consider that the major factor that contribute to the differential infection efficiency between the HS binders and non-HS binders is affinity to the cell surface receptors. Our data suggested that additional host factor(s) contributing to this selection still remain in HS-deficient cells because deletion of HS is not sufficient to prevent the selection of VP1-145G and Q, as already discussed in **lines 465 – 471**. We speculated that negatively charged host molecules contribute to this selection because amino acid substitution at VP1-145 modulates virion surface charge. It may interact with unknown attachment receptor. We are planning to identify such molecules by genome wide CRISPR/Cas9 knock-out (GeCKO) screening using Isehara-E, -G, and -Q viruses in future study. These studies will help to reveal such potential interaction.

Reviewer #2: None

**Part III – Minor Issues: Editorial and Data Presentation Modifications**

Reviewer #1: Fig 5 is difficult to understand

We want to modify Figure 5 to be more understandable. However, we have no idea for the modification because the structure of Figure 5 is quite similar to some other figures (Figure 1, 3, and 4). Please specify the points causing the difficulty if the Reviewer #1 has a good idea. We are willing to modify them.

Reviewer #2: 1. In line 400-403, the authors claimed the difference of infectious efficiency is due to the binding ability of VP1-145E, G, Q virus to HS attachment receptor. In author’s previous research, they demonstrated VP1-145G and Q viruses had higher HS binding ability compared with VP1-145E virus using HS agarose beads. How about the binding efficiency and entry efficiency of these viruses to RD-A and deltaEXT+hSCRB2 cells?

It is difficult to accurately measure the binding/entry efficiency to cultured cells because of various nonspecific factors affecting the results. We previously measured the binding efficiency of the VP1-145 E and G to heparin-agarose beads (Fig 3B of Kobayashi et al. 2018 JVI). The difference of binding affinity is almost the same as that of the infection efficiency; the difference of binding efficiency between VP1-145G and VP1-145E virus is 100 – 300 times measured in the previous study and the difference of infection efficiency in RD-A cells is 238 times in this study. These results suggested that the difference in infection efficiency is largely dependent on difference in binding affinity to HS. We added this discussion in **lines 346-349**.

2. Whether VP1-145E, G, Q mutations directly affect EV71 translation/replication?

We did not test the translation/replication of VP1-145 mutants. Generally speaking, sequence of the capsid region does not critically affect the translation and replication of the virus.

3. Instead of RD-A cells, have the authors tried other cell lines infected with VP1-145E, G, Q viruses? And what are the pattern of viral population?

We conducted the passage experiment using Vero and HeLa-S3 cells and acquired the similar results to the experiment using RD-A cells. We added the data in **Figure 1C-D, 1G-H, and 2A** and explanation for the results in **lines 202-210 and 528-531**.

4. What is the number of mouse per group for testing paralysis and mortality rate?

We used 10 mice per group. We added this in the Materials and Methods section **line 561**.
